# Copper Iodide on Spacer Fabrics as Textile Thermoelectric Device for Energy Generation

**DOI:** 10.3390/ma16010013

**Published:** 2022-12-20

**Authors:** Gabriele Schmidl, Guobin Jia, Annett Gawlik, Philipp Lorenz, Gabriel Zieger, Jan Dellith, Marco Diegel, Jonathan Plentz

**Affiliations:** Leibniz Institute of Photonic Technology (Leibniz IPHT), Albert-Einstein-Straße 9, 07745 Jena, Germany

**Keywords:** copper iodide, thermoelectric, spacer fabrics, smart textiles, Seebeck coefficient

## Abstract

The integration of electronic functionalities into textiles for use as wearable sensors, energy harvesters, or coolers has become increasingly important in recent years. A special focus is on efficient thermoelectric materials. Copper iodide as a p-type thermoelectrically active, nontoxic material is attractive for energy harvesting and energy generation because of its transparency and possible high-power factor. The deposition of CuI on polyester spacer fabrics by wet chemical processes represents a great potential for use in textile industry for example as flexible thermoelectric energy generators in the leisure or industrial sector as well as in medical technologies. The deposited material on polyester yarn is investigated by electron microscopy, x-ray diffraction and by thermoelectric measurements. The Seebeck coefficient was observed between 112 and 153 µV/K in a temperature range between 30 °C and 90 °C. It is demonstrated that the maximum output power reached 99 nW at temperature difference of 65.5 K with respect to room temperature for a single textile element. However, several elements can be connected in series and the output power can be linear upscaled. Thus, CuI coated on 3D spacer fabrics can be attractive to fabricate thermoelectric devices especially in the lower temperature range for textile medical or leisure applications.

## 1. Introduction

The alternative energy generation via thermoelectric effects beside photovoltaics or wind crafts plays an important role in energy recovery from waste heat, such as human body heat, residual heat of engines or thermal heat from big data centers [1,2,3,4,5]. Thermal management systems used in high power energy sources can be supplied for instance to realize best performance for such systems. Furthermore, textile thermoelectrically (TE) active devices represent very cost-effective substrate materials, can be worn on the body and exhibit high flexibility, to also effectively use the heat from shaped surfaces [6,7,8,9]. For the development of textile-based components such as actuators, sensors, flexible energy storage devices or energy generation [10,11,12,13,14,15], simple coating processes such as dipping or laminating of nontoxic but effective working materials are useful for scalable applications or preparation in the textile industry. Flat flexible thermoelectric generators (TEG) with a 2-dimensional architecture could not generate an effective temperature gradient from hot to cold side. One potential design solution is to use the 3D architecture of spacer fabrics. Using spacer fabrics, the challenge is to bring the effective TE material coating on the filaments in-between the surfaces of the fabrics at temperature below 200 °C because of the temperature stability of the yarn materials. This is where physical processes such as vapor deposition or sputtering reach their limits. For aluminum doped zinc oxide (Al:ZnO or AZO) as a n-type semiconductor, produced with atomic layer deposition (ALD) at 180 °C, on spacer fabrics, the function of a TEG could be demonstrated in previous publications [16,17]. 3-D spacer fabrics can be one of a basic textile substrate for thermoelectric energy generators. The spacer filaments coated with TE material represent the material working on the Seebeck effect, which connects the two surfaces of different temperature. In particular, copper iodide (CuI) as a nontoxic material would close a gap as a p-type semiconductor if the yarn could be completely covered during manufacturing.

For using thermoelectricity several material classes with attractive thermoelectric properties were investigated in the last years [18,19,20,21,22]. Good thermoelectric performance can be expected when the dimensionless figure of merit (zT):

zT = S^2^·σ·T/κ (1)

is large. This necessitates a high Seebeck coefficient (S), a high electrical conductivity (σ) and a low thermal conductivity (κ), where S enters squared [23,24], at an absolute temperature (T). Furthermore, the material’s power factor (PF) is defined as:

PF = S^2^·σ (2)

and thus, brings together the thermoelectric power and the conductivity, which is determined via the free charge carriers. Conventional thermoelectric devices consist of n- and p-type thermoelectric legs. Therefore, materials such as tellurides, as Bi_2_Te_3_ or PbTe, half-Heusler compounds, silicides or oxides [25,26,27,28,29] were investigated. However, materials such as carbon nano-tubes (CNT) and poly(3,4-ethylenedioxythiophene):poly(styrenesulfonate) (PEDOT:pss) are also investigated for stretchable TE devices [30] or on 3D structures [31]. Recent works on flexible TEGs for heat harvesting have described ionic thermoelectric (i-TE) hydrogels as a potential material working at low operating temperatures [32,33,34]. In addition, there is a particular interest in nontoxic thermoelectric active materials or materials that are not available in limited quantities, such as tellurium, and that can be easily used to perform energy recovery close to room temperature (RT). This is especially necessary if the application is in the field of medical engineering. In this field toxic bulk materials such as Bi_0.5_Sb_1.5_Te_3_, Bi_2_Te_3_ should be replaced, especially when it comes to body-worn textile elements. Such materials also need to work efficiently near body temperature. Copper Iodide (CuI), represented here for textile application, is used as a p-type semiconductor in various electrical or thermoelectric devices (e.g., transistors, solar cells, transparent electrodes) [35] and shows a good thermoelectric performance also at temperatures lower than 373 K down to room temperature. It is reported as an attractive p-type transparent, nontoxic TE material with a low thermal conductivity of 0.57 W m^−1^ K^−1^ (900–1150 K). A high hole conductivity of >280 S cm^−1^ was observed for the γ-phase of CuI. However, CuI shows phase transitions at higher temperatures to wurtzite (𝛽-CuI) phase and to rock salt (𝛼-CuI), respectively. In literature copper (Cu) is deposited by reactive sputtering on glass or on cellulose paper for example with a subsequent iodine vapor treatment [36,37,38,39,40], which is also often used in industrial routes.

In this study we focus on a complete wet chemical process to prepare CuI on a 3-D spacer fabric consisting of polyester and on the thermoelectric performance of a TE device, with the main consideration being the transferability of the processes to an industrial technology. We analyzed the material, the Seebeck coefficient of the chemical deposited CuI, the electrical conductivity and present a simple multi-TE-element-arrangement for upscaled application.

## 2. Materials and Methods

### 2.1. Coating Processes

The 3-D spacer fabrics provided by ITP GmbH (Weimar, Germany) consist of polyester yarns (approximately 40 spacing filaments per cm^2^) with 4 incorporated copper stranded wires (2 per side), were cut to a size of 2.5 × 2.5 cm^2^ and have a height of 0.8 cm. After cleaning, the overall coating process steps include a copper (Cu) coating of the complete fabric by electroless plating, subsequently a resist protection of the two fabric surfaces and a treatment with iodine vapor in order to convert the Cu coating on the spacing filaments into CuI.

The four-step electroless copper plating process was executed after the formula in [41,42] with slight modifications and is described in detail in a previous publication [16]. It includes the cleaning of the textile, sensitization of the textile, activation by Palladium (Pd) catalyst deposition and dip into an electroless copper plating bath. The textile is cleaned by acetone with the help of sonication for about 10 min, and subsequently rinsed with isopropanol and treated in a plasma cleaner for 10 min. The sensitization bath contains 5 g/L SnCl_2_ (Sigma Aldrich, Taufkirchen, Germany) added with 40 mL/L of 38 wt% HCl (J. T. Baker, by Fisher Scientific GmbH, Schwerte, Germany) and the activation bath 0.25 g/L PdCl_2_ (Sigma Aldrich) and 2.5 mL/L of 38% HCl (J. T. Baker). The treated textile is then placed in an electroless plating bath for 5 min. Longer times result in thicker layers.

After Cu deposition the two surfaces are protected by a resist AZ5214E and the sample is backed in the oven at 80 °C for 10–15 min. This method is applied on both surfaces after each other.

The sample is then exposed to iodine vapor in a furnace. This is carried out in a lockable glass jar.

2Cu + I_2_ → 2 CuI (3)

The conversion takes about 15 h overnight at 40 °C. Throughout the process, the free Cu stranded wires should also be protected by a resist.

The resist is then removed for performing the thermoelectric measurements. Both surfaces can be coated subsequently with conductive silver to increase the electrical conductivity and to lower the contact resistance, and with this to increase the TE efficiency. This can be carried out, for example, by dipping or painting.

For the temperature dependent conductivity measurements and material analysis, Cu was also deposited on glass substrates (25 × 25 × 1.1 mm) by the wet chemical process as well as by dc-sputtering for comparison and also converted in iodine vapor to CuI.

### 2.2. Material Characterization

For material characterization scanning electron microscopy (SEM, FE-SEM JSM-6700F; JEOL Ltd., Tokyo, Japan) images and microanalytical measurements (EDX) were performed. The EDX spectra were taken using a Silicon Drift Detector (SDD) XFlash 5130 from BRUKER Nano GmbH, Berlin, Germany. The energy of the exciting electrons was set to 12 keV and the accumulation live time was 500 s. Beyond that X-ray mappings were performed to illustrate the element homogeneity of the fabricated CuI films. In order to prevent charging effects in the SEM, a 5 nm thick carbon layer was deposited on the sample before analysis. The crystalline analysis of the CuI layers on the polyester fiber was investigated by X-ray diffraction (XRD, Panalytical X’Pert Pro) with Cu-Kα_1,2_ (Kα1: 1.5406 Å) radiation.

### 2.3. Temperature Dependent Electrical Conductivity of CuI Films

The electrical conductivity was determined using the 4-point method in linear configuration on samples with wet-chemically deposited Cu (200 nm thick CuI) as well as with sputtered Cu (1.1 µm thick CuI) alternatively on 2.5 × 2.5 cm^2^ glass substrates (1 mm thickness) and subsequent iodization.

For the temperature dependent measurements using the sputtered Cu seed layer, the tips have had a spacing of s = 2 mm which could be varied. These measurements were made with a FormFactor PA200 probe system with a heatable sample stage, while using a Yokogawa GS200 as current source and a Kethley2000 digital multimeter to determine the potential difference. The preset probe temperature in the range of room temperature to 80 °C was reached after approx. 1 min and the measured value was recorded after approx. 10 min, after a stable measured value had been obtained. A second measurement setup only for RT measurements uses a 236 Source Measure Unit from Keithley.

In general, voltages were measured between the inner tips, while at the outer tips a current from 2 to 50 µA was applied. Geometry factors were determined and applied to accurately determine the resistance values [43,44]. The following equation was used to calculate the electrical conductivity:

σ = 1/ρ = 1/((V/I)·(π/ln2)·d·f_1_·f_2_·f_3_) (4)

In this case, (V) is the voltage, (I) is the current and (d) is the thickness of the layer. Additionally, we need f_1_, f_2_ and f_3_ as correction factors which consider the layer thickness to the spacing of the tips, refer to the distance of the tips to the sample edges or to the sample shape. In our case for d << s, f_1_ was determined to 1 as well as f_2_. f_3_ is 0.95 for the squared sample a = b = 2.5 cm.

### 2.4. Thermoelectric Measurement Setup for Fabrics

**Figure 1:**
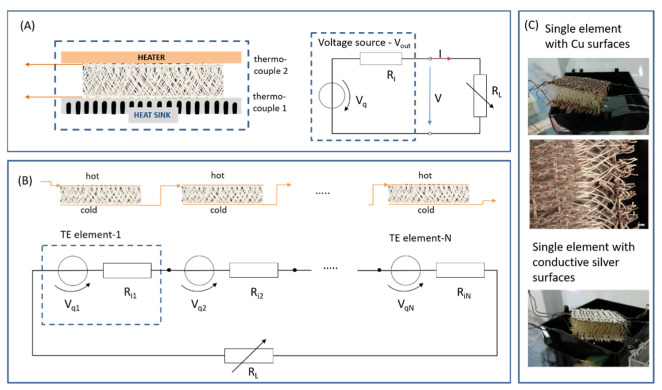
(**A**) Schematic description of a single textile element setup and equivalent circuit diagram for TE measurements, (**B**) circuit of several elements for applications, (**C**) CuI coated 3D fabrics with copper top and bottom surfaces and additional with a conductive silver coating on the surfaces, photos and microscope image (digital microscope KEYENCE VHX7000) in the middle.

The Seebeck coefficient was measured with a setup, where the spacer fabric was fixed between a heater with a thin glass substrate on the hot side and a metal heat sink on the cold side (Figure 1). The temperature on the “cold side” was kept nearly constant at room temperature (22–23 °C) and the heater was used to warm up the “hot side”. It was always waited until a stable temperature regime has been established. The temperatures on both hot and cold side of the fabric surfaces were measured by thermocouples PT100, which were connected with a two-channel digital thermocouple thermometer (Omega Engineering), and for determining the voltage and current, an HP 3457A multimeter was used. The values of voltage and current measurements were carried out with discretely adjustable load resistors from 1 to 1000 Ω, which were integrated into the electrical circuit. These values were then used to create the current (I)-voltage (V) characteristics, to determine the open-circuit voltage (V_OC_ for I = 0) and short-circuit current (I_SC_ for V = 0) for each temperature difference (ΔT). This is due to the method of a power adjustment (Figure 1A). Measurement uncertainties exist for the determination of very small voltages and, in particular, for temperature measurements. In addition, the stability during handling and measuring of the samples must be guaranteed. For the circuit of several single textile elements, a series connection was used in accordance with photovoltaic systems, considering the hot and cold sides (Figure 1B). These connected elements are used to show how it works on warm human skin and on a hot surface to generate energy. Figure 1C shows the CuI coated fabrics.

## 3. Results and Discussion

### 3.1. Material Investigations

The process of the electroless Cu plating has a great advantage in the field of wet chemical processes because it can be performed on nonconducting substrates such as ceramics, polymer, or biomaterials [16,42]. However, using the spacer fabrics made of polyester (PE) the main focus must be on complete coating of the spacer filaments and on the adhesion of the layer. Therefore, we used isopropanol cleaning and plasma functionalization within air atmosphere. This physical functionalization by plasma is preferable when metallic contact threads have already been woven into the fabric and is a more suitable process for industrial use. The deposited copper forms a smooth surface on the polyester yarns, dependent on the reaction time.

**Figure 2:**
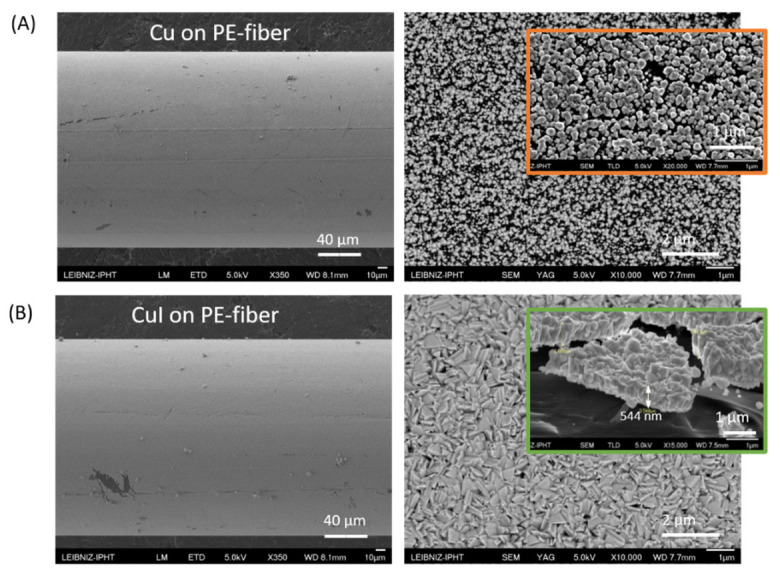
(**A**) Copper on polyester fiber 5 min deposition time, left: SE image, 5 kV, ×350, right: BSE image, 5 kV, ×10k. (**B**) Copper iodide coated polyester fiber, left: SE image, 5kV, ×350, right: BSE image, 5 kV, ×10k. Insets: thin film and surface structure of Cu and CuI. The BSE (backscattered electrons; Z-contrast) micrographs show no evidence of inhomogeneities.

The PE fiber surface coated with Cu for 5 min, seen in Figure 2A, is small-grained. Coating the two fabric surfaces with resist prevents the Cu on the surfaces and the interwoven Cu stranded wires from also being converted in the iodine vapor to CuI. The sheet resistance of the Cu surfaces after removing the resist was determined to be below 1 Ω. The resistance from surface to surface after iodization was obtained of about 2 kΩ only with this copper surface coating. An additional conductive silver coating on top of both copper coated surfaces lowers the resistance from surface to surface to about 400 Ω. We suppose that the conductive silver increases the electrical conductivity in plane and lower the contact resistance. The resistances were measured diagonally across the surface or the height. For a 5 min Cu deposition on a PE fiber and subsequent transformation to CuI, a film thickness of about 500 nm was determined (Figure 2B). It was observed in all experiments, on a fiber and on glass, that the film is closed, and the thickness based on different thick copper seed films increases of approximately 4–5 times after iodization.

**Figure 3:**
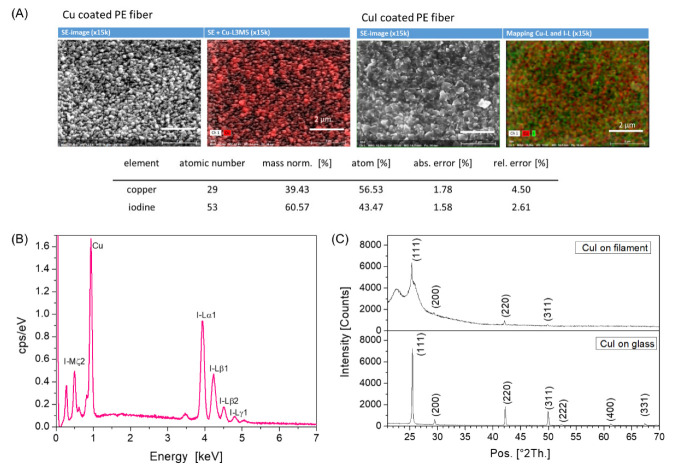
(**A**) EDX mapping of a coated PE fiber—SE image and Cu-L und I-L mapping, 12 kV, magnification: ×15,000; the EDX mapping shows both Cu-rich and J-rich spots. (**B**) EDX spectrum of CuI taken from the PE fiber, 12 kV, 500 s; (**C**) XRD spectra of 500 nm thick CuI on a PE fiber (ref. card ICDD 01-083-1108) in comparison to 800 nm CuI with sputtered Cu on a glass substrate (ref. card ICDD 01-085-1326).

The spacer fabrics were chosen to be widely woven (approximately 40 filaments per cm^2^), so that the iodine vapor can penetrate well into all interstitial spaces and a complete transformation of the copper can assume on all filaments. In order to show, how the iodine vapor transforms the copper and which phase of the CuI is to be found we applied EDX and XRD analysis (Figure 3) on coated single fibers and glass substrates. The EDX mapping reflects the transformation of the Cu over the entire surface (Figure 3A) and the spectrum can be used to determine the composition of the layer (Figure 3B). From these studies, a composition of 57% atom copper and 43% atom iodine is obtained. It can be concluded that the major fraction of the copper has been converted. However, this can be seen more precisely by crystal analysis using XRD (Figure 3C). This method allows a statement about the exact composition of pure materials and compounds and the crystal structure or parameters. The main peaks of Cu(I)iodide on a filament are found at 25.3° (111), 42.1° (220) and 49.8° (311) as well as a smaller one at 29.3° (200). Distinct reflexes from pure copper at 43.2° (111) and 50.2 (200) are not to be seen. The background originates from the substrate. The forming Cu(I)iodide crystallizes in the cubic crystal system and the crystal structure corresponds to the zinc blende structure. The stable zinc blende phase is found below 350 °C, which is a typical p-type semiconductor, changes to wurtzite (𝛽-CuI) phase between 350 °C and 380 °C and to rock salt (𝛼-CuI) at higher temperatures [36,45]. An almost complete conversion to CuI was not only demonstrated for a thin copper layer on the filaments, but in comparison also confirmed using a 200 nm thick copper layer on a glass substrate by XRD.

### 3.2. Thermoelectric Investigations

In order to characterize the thermoelectric parameters (Seebeck coefficient (S) and output power (P_out_) of the material using a single element, means one leg of a bigger module (see Figure 1A,B), the temperature of the “hot side” was varied from room temperature 295 K to 360 K. The temperature on the “cold side” was kept constant at room temperature. This small range was chosen, because this is the target temperature range for textile applications. Furthermore, the maximum temperature resistance of polyester up to 180 °C (https://www.siltex.de/temperatur.html (accessed on 10 August 2022)) must be considered. The woven metallic contact threads in the fabric surfaces were used to measure the generated voltages and currents.

**Figure 4:**
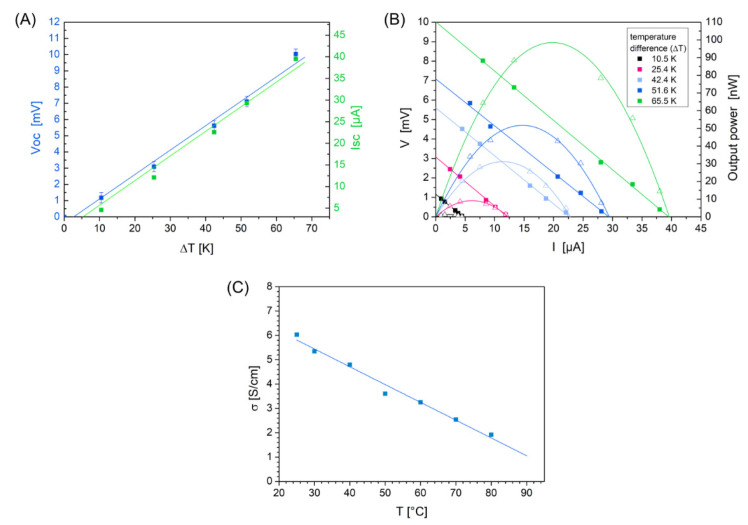
(**A**) Open-circuit voltage (V_OC_) and short-circuit current (I_SC_) versus temperature difference between hot and cold surface, a small bias voltage of −0.01 mV was obtained. Note the scaling of the I_SC_ axis for better visualization. S = 150 µV/K was determined by the slope of the curve V_OC_(︵T). (**B**) I-V characteristics with load resistances of 10, 50, 100, 500, 1000 Ω. Output power for a single textile element, (**C**) electrical conductivity was measured temperature dependent. Sputtered Cu layer was used for transformation to CuI (linear fit for visualization).

The determined open-circuit voltage (V_OC_ for I = 0) and short-circuit current (I_SC_ for V = 0) show a linear increase with increasing temperature difference ︵T (Figure 4A). Using the I-V characteristics in Figure 4B, the maximum output power (P_max_) can be calculated corresponding to:

P_max_ = ¼ V_OC_·I_SC_ (5)

with V_OC_ = S·︵T for each measured temperature difference ︵T. Equation (5) is valid since P_out_ is defined by:

P_out_ = (V_OC_)^2^ R_L_/(R_L_ + R_i_)^2^ (6)

where R_L_ is the load resistance and R_i_ the internal resistance of the thermoelectric leg or module and in case of P_max_ both resistances R_L_ and R_i_ are obtained to be equal [5,26,39]. A single TE element with a conductive silver top layer generates in our study a maximum output power of P_max_ = 99 nW at ︵T = 65.5 K or 1.3 nW at ︵T = 10.5 K (Figure 4B). At the same temperature difference of 60 K, we achieved a P_max_ value of 65 nW when only the Cu coating was used on the surfaces, or 76 nW with the conductive silver coating in comparison. This comparison shows that with conductive silver the maximum power can be increased based on a higher short-circuit current, because conductive silver increases the electrical conductivity in plane and lower the contact resistance.

For S the study yield values between 112 µV/K and 153 µV/K for temperatures from 32 °C to 87.5 °C on the “hot side” of a single element. The Seebeck coefficient is determined by the open-circuit voltage V_OC_ from Figure 4B and the measured temperatures on the two surfaces of the spacer fabric (S = V_OC_/︵T). Assuming a linear relationship between V_OC_ and ︵T shown in Figure 4A, S can be calculated from the slope of the linear fit curve with S = d(V_OC_)/d(︵T). The resulted value for S amounts 150 µV/K (standard error +/−8 µV/K) and is influenced by the accuracy of the determination of V_OC_ derived from Figure 4B (maximum error ︵V_OC_ = +/−0.3 mV) and the accuracy of the temperature measurements (︵(︵T) = +/−1 K). Furthermore, a small bias voltage of −0.1 mV was observed. Comparing the Seebeck values with those of [36] (S = 170 µV/K) at 300 K for a hole concentration of 1 × 10^20^ cm^−3^ or of [40] (S = 140 µV/K) at 300 K, our measured values are slightly lower. This may be related to different fabrication methods, reactive sputtering, sputtering with subsequent chemical iodization, or as here, wet chemical electroless deposition on PE fibers followed by chemical iodization, and therefore related to the associated layer properties for the copper or copper iodide. Furthermore, a spacer fabric presents a completely different geometry than flat layer surfaces.

Figure 4C demonstrates the measured electrical conductivity of the prepared CuI dependent on the temperature. An approximately 180 nm thick sputtered Cu film on glass, measured by a surface profiler (Tencor P-10), was used for iodization which results in a 1.1 µm thick CuI film. The electrical conductivity obtained ranges from 2 to 6 S cm^−1^. These values are about 4 times lower than in the literature [40,46]. The slightly lower values may be due to the manufacturing process and layer thickness determination. Using a wet chemical deposited thin copper layer which results in an approx. 200 nm CuI film, the electrical conductivity shows a value of 5.6 S cm^−1^ at room temperature and possesses a similar value to the sputtered Cu with 6.0 S cm^−1^. The relationship shown in Figure 4 was therefore used for the further calculations of PF. The microstructure is of particular importance, since scattering effects cannot be neglected. For a complete electrical material characterization and to characterize the semiconducting properties, it is useful to measure also at low temperatures and to determine charge carrier concentration (N) and mobility (µ), since for p-type semiconductors with negligible electron conduction, the conductivity value can be obtained from σ = q·(N·µ). This more detailed investigation is to be realized with future investigations.

The power factor as one part of TE characterization is determined by the product of Seebeck coefficient squared and the electrical conductivity and yields values for example of PF = 6.6 × 10^−6^ W m^−1^ K^−2^ near room temperature (RT) (Table 1). The comparison with the values from literature (3.59 × 10^−4^ W m^−1^ K^−2^ [36]) shows a smaller PF, nearly two magnitudes. This can also be explained by the comparatively different production method of the CuI on fabrics and thus also with the different charge carrier densities but also with the somewhat smaller Seebeck coefficient near RT.

Optimization of the electrical and thermoelectric properties can be achieved by layer optimization with respect to processes and thickness parameters or, as described in [46], by a subsequent annealing process. In [46] is presented how an additional annealing process affects the electrical and structural properties of CuI and how the thermoelectric properties can be improved by changing the native defect structure in the lattice. This influence was not considered here, but will be a possible subject of further investigation for temperatures below 200 °C using textiles.

### 3.3. Electrical Interconnection of Several Single Elements

**Figure 5:**
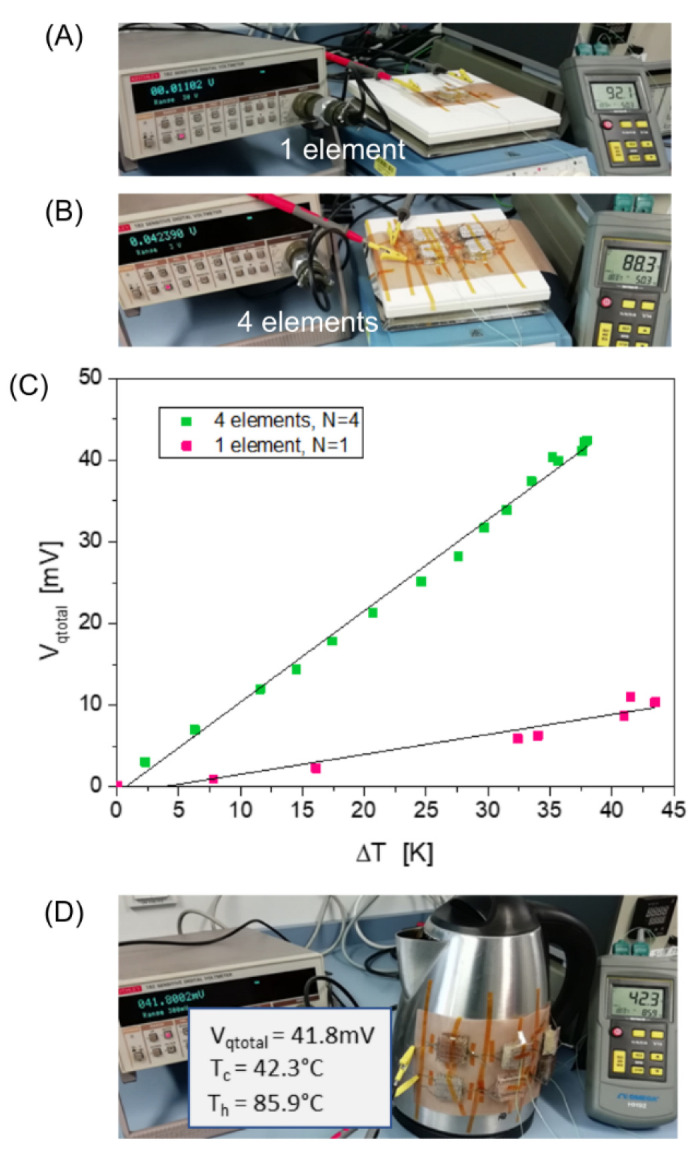
Output voltage V_qtotal_ dependent on the number of interconnected elements N, (**A**) image of a measurement setup with one TE element on a hot plate, (**B**) with four TE elements on a hot plate. Spacer fabrics with conductive silver surfaces are used. N = 4, R_itotal_ = 2 kΩ. (**C**) Total output voltages in comparison. (**D**) Example of the everyday life, a kettle with 6 textile elements with Cu surfaces.

In order to operate small electronic components with textile TEGs two routes are possible and can be combined. On one hand an energy harvesting and storage is possible, if a higher energy is only needed in a short time period. On the other hand, the voltages and currents or powers can be increased by an electrical interconnection of single TEG elements. This means that a textile TEG made of CuI can achieve a higher power range by interconnecting several individual elements. For the electrical interconnection, however, care must be taken to ensure that each textile element has low and similar resistance parameters. Since the example circuit presented in Figure 5 is not a standard TE device with p- and n- semiconductors connected in series, the surfaces of the individual elements are properly alternating connected, as shown in Figure 1B.

In a series circuit, different resistances (R_iN_) cause different voltage drops (V_qN_). The partial voltages behave similar to the corresponding resistances and the voltages as well as the resistances of each element are accumulated. Therefore, in Figure 5A, an increase in overall voltage can be seen when 4 elements (N = 4) are used instead of one element (N = 1). Furthermore, the summed voltages for a fixed number of single elements increases linearly with the temperature difference. The current through the series circuit, for example, can be determined by the overall voltage V_iqtotal_ and the overall resistance R_itotal_, since it flows through each element with the same amount. With these 4 elements a power of 0.88 µW can be achieved. An optimal case to construct a TE fabric array is to use elements with low and similar partial resistances Ri_N_ to higher the current. Figure 5B represents an example from everyday life using a kettle.

These application-oriented experiments show that the unfavorable flexibility of spacer fabrics, which is often mentioned due to 3D warping, can be overcome by connecting individual textile elements to form an overall construct with series connection. The size of this overall construct is thus also adjustable. Furthermore, in contrast to a conventional TE device, the use of a single TE material has the advantage that only one TE material (p- or n-material) with optimally similar performance parameters has to be found. Encapsulation of the textile single element e.g., with silicone can further increase stability of the hole device and the adhesion of the CuI film. When depositing CuI in chemical-only process steps, it is generally important to ensure a high Seebeck coefficient, good adhesion and low reproducible resistance to increase efficiency. Another limiting factor is thermal radiation through the spacer fabric. However, this can be countered by dissipating the heat from the outer surface, for example with heat conducting foils. Future work will pursue these requirements.

## 4. Conclusions

All results show that CuI has a great potential as a non-toxic thermoelectric material also for use in textile applications. This material can be used for low-power applications, for example as a small body-heat-powering for wearable devices or small LEDs. Using spacer fabrics and wet chemical processing the production of TE elements is industrially feasible and allows upscaling.

CuI can be adhesively deposited on polyester fibers at low temperatures with a wet chemical process and shows a high Seebeck coefficient at temperatures near room or body temperature. This is essential for industrial production and human or medical applications. Spacer fabrics as a textile TE element can be effectively used on flat or bended surfaces and single textile elements generate for example an output power of 99 nW at ︵T = 65.5 K or 1.3 nW at ΔT = 10.5 K. Individual CuI spacer fabric elements can be connected to each other to increase the output voltages or the power. In this way, an increase in output voltage from 1.6 mV to 10.4 mV at ︵T = 10 K demonstrates the effect of a series circuit.

The efficiency of a TE device is determined by the power factor, the thermal conductivity and the absolute temperature. However, it is important to note, that the efficiency using fabric architectures for human application is only one point for consideration. Characteristics such as comfort, flexibility, stability over time, toxicity, a low temperature range, or processability are crucial on the other side. In the further progress of this work, the properties of CuI on textile fabrics are to be optimized and an encapsulation of the textile single elements must be developed to further improve the output parameters, the stability and the reproducibility.

## Figures and Tables

**Table 1:** The summarized measured and calculated values of open-circuit voltage, short-circuit current, maximal power output, Seebeck coefficient, electric conductivity and power factor for different temperatures (see Figure 4).

V_OC_ [mV]	I_SC_ [µA]	P_max_ [nW]	T_abs_ [K]	S [µV K^−1^]	σ [S m^−1^]	PF [W m^−1^ K^−2^]
1.2	4.6	1.4	305	112	530	6.6 × 10^−6^
3.1	12.1	9.4	320	122	418	6.2 × 10^−6^
5.6	22.6	31.6	337	133	295	5.2 × 10^−6^
7.1	29.3	52.0	346	138	230	4.3 × 10^−6^
10.0	39.5	98.7	360	153	127	2.9 × 10^−6^

## Data Availability

The raw data are stored at the Leibniz Institute of Photonic Technology and are available there.
